# A new heparan sulfate from the mollusk *Nodipecten nodosus* inhibits merozoite invasion and disrupts rosetting and cytoadherence of *Plasmodium falciparum*

**DOI:** 10.1590/0074-02760190088

**Published:** 2019-06-06

**Authors:** Marcele F Bastos, Letusa Albrecht, Angélica M Gomes, Stefanie CP Lopes, Cristina P Vicente, Rodrigo PM de Almeida, Gustavo C Cassiano, Roberto JC Fonseca, Claudio C Werneck, Mauro SG Pavão, Fabio TM Costa

**Affiliations:** 1 Universidade Estadual de Campinas, Departamento de Genética, Evolução, Microbiologia e Imunologia, Laboratório de Doenças Tropicais, Campinas, SP, Brasil; 2 Fundação Oswaldo Cruz-Fiocruz, Instituto Carlos Chagas, Curitiba, PR, Brasil; 3 Cleveland Clinic Lerner Research Institute, Department of Biomedical Engineering, Cleveland, OH, USA; 4 Fundação Oswaldo Cruz-Fiocruz, Instituto Leônidas e Maria Deane, Manaus, AM, Brasil; 5 Universidade Estadual de Campinas, Departamento de Biologia Estrutural e Funcional, Campinas, SP, Brasil; 6 Universidade Federal do Rio de Janeiro, Centro de Ciências da Saúde, Instituto de Ciências Biomédicas, Hospital Universitário Clementino Fraga Filho, Laboratório de Tecido Conjuntivo, Rio de Janeiro, RJ, Brasil; 7 Universidade Estadual de Campinas, Departamento de Bioquímica e Biologia Tecidual, Campinas, SP, Brasil; 8 Universidade Federal do Rio de Janeiro, Instituto de Bioquímica Médica Leopoldo de Meis, Programa de Glicobiologia, Rio de Janeiro, RJ, Brasil

**Keywords:** glycosaminoglycan, severe malaria, adhesion, antimalarial, adjuvant therapy

## Abstract

**BACKGROUND:**

Despite treatment with effective antimalarial drugs, the mortality rate is still high in severe cases of the disease, highlighting the need to find adjunct therapies that can inhibit the adhesion of *Plasmodium falciparum*-infected erythrocytes (Pf-iEs).

**OBJECTIVES:**

In this context, we evaluated a new heparan sulfate (HS) from *Nodipecten nodosus* for antimalarial activity and inhibition of *P. falciparum* cytoadhesion and rosetting.

**METHODS:**

Parasite inhibition was measured by SYBR green using a cytometer. HS was assessed in rosetting and cytoadhesion assays under static and flow conditions using Chinese hamster ovary (CHO) and human lymphatic endothelial cell (HLEC) cells expressing intercellular adhesion molecule-1 (ICAM1) and chondroitin sulfate A (CSA), respectively.

**FINDINGS:**

This HS inhibited merozoite invasion similar to heparin. Moreover, mollusk HS decreased cytoadherence of *P. falciparum* to CSA and ICAM-1 on the surface of endothelial cells under static and flow conditions. In addition, this glycan efficiently disrupted rosettes.

**CONCLUSIONS:**

These findings support a potential use for mollusk HS as adjunct therapy for severe malaria.

*Plasmodium falciparum* accounts for most cases of severe and lethal malaria, causing 435,000 deaths per year, especially in children under five years old.^1^ Malaria affects approximately 25% of pregnant women living in malaria endemic regions and is responsible for up to 20% of maternal deaths.^1^ Although several factors contribute to the virulence of this species, binding of *P. falciparum*-infected erythrocytes (Pf-iEs) to the vascular endothelium (cytoadherence) plays a major role.^2^ In addition, Pf-iEs can also adhere to infected (auto-agglutination) and uninfected erythrocytes (rosetting). These events may lead to occlusion of the microvasculature of vital organs, contributing to the pathogenesis of severe malaria.^2^ ^,^ ^3^

Although current malaria therapy kills parasites quickly, it has already been shown that non-viable Pf-iEs retain the ability to cytoadhere in the first 24 h after administration of drugs, including artesunate,^4^ which probably contributes to the high mortality observed in the first hours of hospital admission even after the recommended treatment.^5^ Based on this, the use of adjunct therapies has been proposed. Due to its anticoagulant properties, heparin was used in the past as adjunctive treatment for severe malaria, but its use is hindered by side effects, including bleeding events.^6^ Previous studies have shown that other sulfated polysaccharides with improved safety profiles are also able to prevent cytoadherence of *P. falciparum* to several host receptors, inhibit merozoite invasion, and disrupt rosettes.^7^ ^,^ ^8^ ^,^ ^9^ ^,^ ^10^

Previously, we described a new heparan sulfate (HS) isolated from the mollusk bivalve *Nodipecten nodosus*. This unique HS is formed by repeating disaccharide units of β-d-glucuronic acid 1→4 *N*-acetyl-α-d-glucosamine containing a rare sulfation pattern on carbon 2 or 3 of the glucuronic acid units [Supplementary data (Fig. 1)]. The mollusk glycan exhibits antithrombotic activity without inducing bleeding. Furthermore, experiments in vivo demonstrated that HS attenuates hematogenous metastasis, thrombosis and inflammation.^11^ ^,^ ^12^ Here, we have examined the antimalarial activity of this compound and its effects on adhesion to the host receptors chondroitin sulfate A (CSA) and intercellular adhesion molecule-1 (ICAM-1), as well its ability to disrupt rosettes.

## MATERIALS AND METHODS

*Mollusk HS* - Several specimens of the marine mollusk *N. nodosus* were kindly provided by the Instituto de Ecodesenvolvimento da Baía da Ilha Grande ― IEDBIG (Rio de Janeiro state, Brazil) and used for extraction of HS. The HS purification protocol was described elsewhere.^11^

*Parasite culture and growth inhibition* - Rosetting FCR3S1.2 and non-rosetting FCR3 strains were cultivated in fresh type O^+^ human erythrocytes with complete parasite medium and kept at 37ºC in 5% CO_2_ and 5% O_2_.^13^ To achieve a synchronous culture, consecutive treatments with a 5% solution of d-sorbitol were applied at 48-h intervals. The effects of mollusk HS on specific stages of *P. falciparum* development was assessed by growth inhibition assay.^14^ Briefly, after synchronization, young trophozoites (~ 24-h old) or ring forms (~ 3-h old) were incubated in 96-well plates with different concentrations of mollusk HS or heparin for 24 h or 48 h. After incubation, parasite growth inhibition was measured by SYBR green using a cytometer.

*Static cytoadhesion assay* - FCR3 parasites that adhered to ICAM1 (Pf-iEs^ICAM-1^) or CSA (Pf-iEs^CSA^) were selected by panning on Chinese hamster ovary (CHO)-ICAM1 or human lymphatic endothelial cells (HLECs) cells, respectively, as described before.^7^ Then, Pf-iEs^CSA^ and Pf-iEs^ICAM-1^ were incubated over confluent HLEC or CHO-ICAM1 monolayers for 1 h alone or in the presence of several concentrations of HS. After extensive washing to remove non-adherent Pf-iEs, the slides were fixed and stained with Giemsa, and the percentage inhibition relative to a control was determined under the microscope.^7^

*Flow-based cytoadhesion assay* - The ability of the mollusk HS to desequester Pf-iEs was assessed using flow-based cytoadhesion assays, as described previously.^15^ Briefly, 5 × 10^5^ Pf-iEs^CSA^ were added to the culture slides and allowed to cytoadhere statically. After 1 h, culture slides were mounted in a flow chamber system through which cytoadhesion medium (control) or HS (100 µg/mL) was passed under different shear stresses. The remaining Pf-iE adherents were counted in 30 randomly chosen fields.

*Rosetting assay* - FCR3S1.2 parasites were treated with different concentrations of mollusk HS or heparin at 37ºC for 1 h. Parasite medium was used as control. Then, parasites were stained with acridine orange and the rosetting formation was determined under a microscope. A rosette was scored when a parasitized cell was bound to at least two non-infected erythrocytes.^8^

*Statistical analysis* - Statistical significance was determined using one-way analysis of variance or Student’s t test for parametric data. Kruskal-Wallis and post hoc tests or Mann-Whitney’s U test were used for non-parametric data. All statistical analyses were performed using Prism version 5.02 (GraphPad, USA), and values were considered significant when p < 0.05.

## RESULTS AND DISCUSSION

**Fig. 1:**
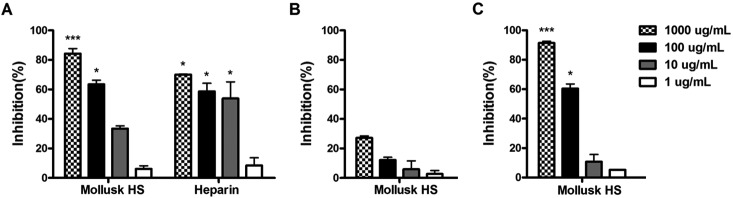
mollusk heparan sulfate (HS) inhibits *Plasmodium falciparum* growth and merozoite invasion. Ring synchronized *P. falciparum*-infected erythrocytes (A and B) or late-stage forms (trophozoites) (C) cultured at 0.8% parasitemia and 2% hematocrit were incubated at 37ºC for 48 h (A) or 24 h (B and C) with increasing concentrations of mollusk HS or heparin. The percentage of growth inhibition was calculated relative to control values (parasites were grown in RPMI 1640 medium), and the results are expressed as the means ± SD of triplicates. *p < 0.05; ***p < 0.001 (Kruskal-Wallis and Dunn’s post hoc test).

*Effects of mollusk HS on P. falciparum growth* - To evaluate the antimalarial activity of the mollusk HS, we analyzed *P. falciparum* growth for 48 h using the fluorescence-based SYBR green assay. Like heparin, the mollusk HS inhibited parasite growth in a dose-dependent manner (Fig. 1A). To investigate whether the mollusk HS inhibited merozoite invasion or intracellular parasite development, synchronized ring-stage parasites were cultured for 24 h in the presence of the mollusk HS. As shown in Fig. 1B, ring maturation rates were not significantly reduced even at the highest HS concentration (1000 µg/mL). On the other hand, when synchronized late-stage forms were incubated with the mollusk HS, a significant decrease in parasite count was observed compared with untreated controls at 24 h after treatment (Fig. 1C). This result suggests that mollusk HS blocked merozoite invasion (or schizont rupture) in a dose-dependent manner and reached up to 91% inhibition at the highest dose (1000 μg/mL). It has been demonstrated that growth inhibition of *P. falciparum* by other sulfated polysaccharides, such as heparin, sulfated fucans, and fucosylated chondroitin sulfate, is due to inhibition of invasion of red blood cells by merozoites.^7^ ^,^ ^9^ The exact mechanisms by which these compounds could affect merozoite invasion are still unclear, although a study has shown that heparin binds to several erythrocyte-binding *P. falciparum* proteins, including ligand molecules that are essential for moving junction complex formation.^16^

*Effects of mollusk HS on P. falciparum adhesion interactions* - Currently, intravenous artesunate is recommended as the first-line therapy for patients with malaria complications. The pathologic hallmark of severe *P. falciparum* malaria is the sequestration of parasitized erythrocytes in various tissues due to interactions mediated mainly by *P. falciparum* erythrocyte membrane protein 1 (PfEMP-1) with several host receptors on endothelial (cytoadhesion) or normal red blood cells (rosetting). Importantly, in vitro cytoadherence of Pf-iEs continues for several hours after parasite death by antimalarials as a result of slow degradation of PfEMP-1 on the surface of Pf-iEs,^4^ which suggests that the high mortality after hospital admission despite effective treatment may be due to this persistent parasite adhesion. Therefore, the use of adjunct therapies is urgently needed.

Here, we selected FCR3 parasites for a CSA- or ICAM-1-binding phenotype. CSA is mostly expressed in placental trophoblasts and is an important receptor for sequestration of Pf-iEs, which seems to be a key feature in the pathogenesis of placental malaria.^17^ In turn, the role of ICAM1 in the sequestration of Pf-iEs in the microvasculature of other organs, such as the brain, remains elusive, probably due to the high number of host receptors involved in parasite adhesion. However, there is abundant evidence that supports a critical role of this receptor associated with disease severity (as reviewed in^18^).

**Fig. 2:**
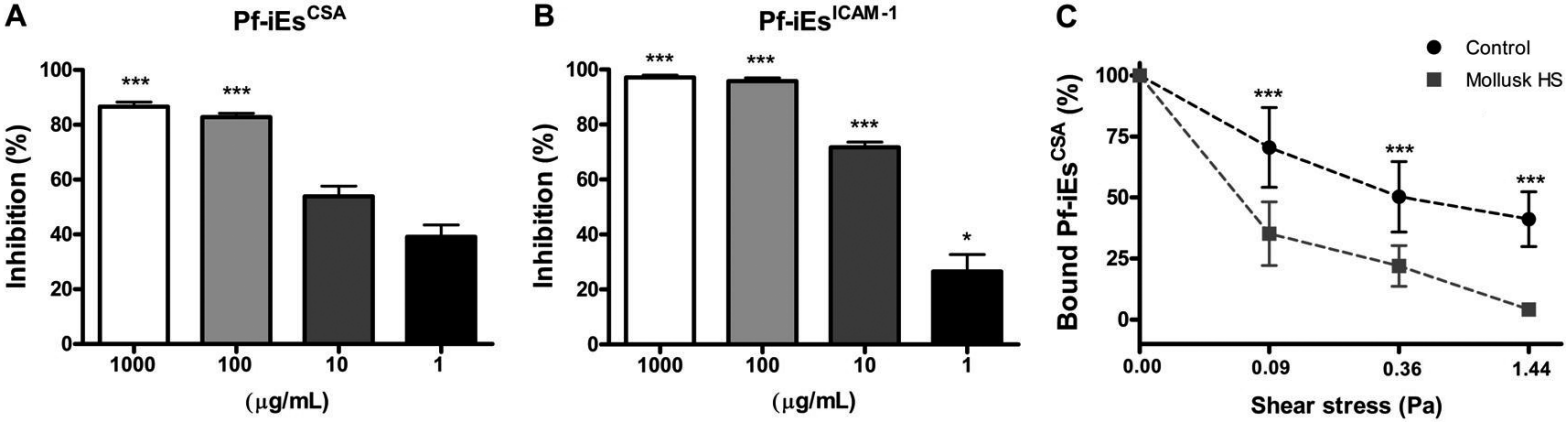
the effect of mollusk HS on cytoadhesion of *Plasmodium falciparum*. Cytoadhesion of FCR3 strain after incubation of chondroitin sulfate A (CSA)- (A) or intercellular adhesion molecule-1 (ICAM1)-binding (B) *P. falciparum*-infected erythrocytes (Pf-iEs) with different concentrations of heparan sulfate (HS) is shown under static conditions. Mollusk HS is also capable of reversing binding of Pf-iEs^CSA^ on human lymphatic endothelial cells (HLECs) under flow conditions with increasing shear stress (C). Data are expressed as percentages relative to control. Data are representative of three independent assays with error bars indicating ± SD. *p < 0.05; ***p < 0.001 (Kruskal-Wallis and Dunn’s post hoc test).

**Fig. 3:**
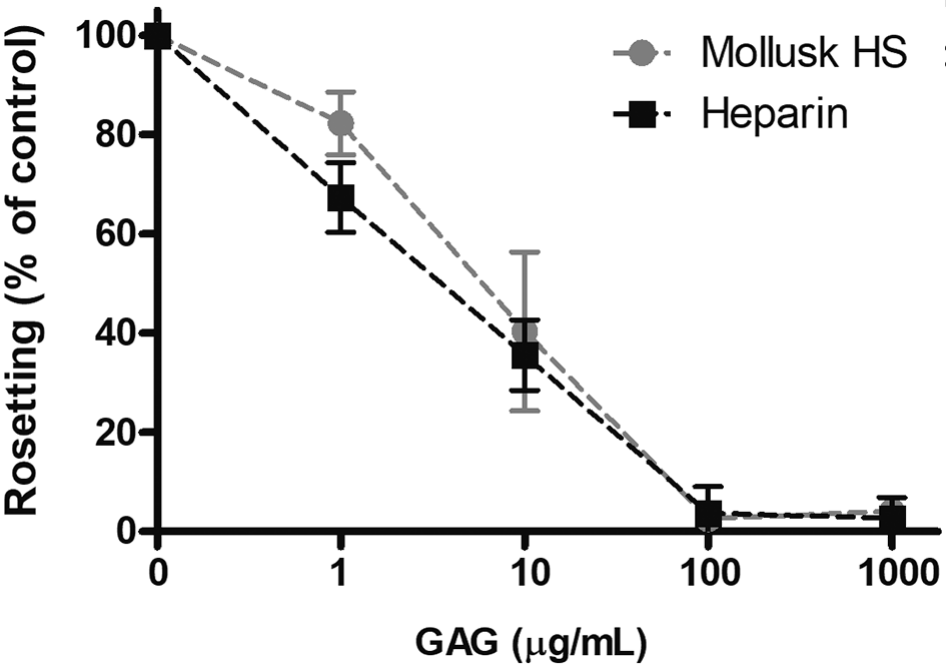
mollusk heparan sulfate (HS) and heparin disruption of *Plasmodium falciparum* rosettes after incubation for 1 h is shown at the indicated concentrations of each compound. Data are expressed as percentages relative to control (no compound). Data are shown as the mean of two independent experiments with error bars indicating ± SD. Glycosaminoglycans (GAG).

Based on the ability of heparin and other glycoconjugates in inhibiting *P. falciparum* cytoadhesion to endothelial cells and rosette formation, we evaluated the effects of the mollusk HS on parasite adhesion events in three ways. (i) We showed that HS inhibited cytoadherence even at the lowest dose tested (1 µg/mL), and reached 86% and 100% inhibition of Pf-iEs^CSA^ (Fig. 2A) and Pf-iEs^ICAM-1^ (Fig. 2B), respectively, at the highest dose (1000 µg/mL); (ii) having demonstrated the ability of HS to inhibit the cytoadherence of Pf-iEs under static conditions, we investigated whether the compound was able to reverse cytoadhesion of Pf-iES^CSA^ to HLECs under flow conditions. As shown in Fig. 2C, 100 µg/mL HS significantly reversed Pf-iEs^CSA^ adhesion compared with controls for all shear stresses tested. Only 4.1% ± 2.6% of parasites remained attached to HLECs at the highest shear stress, a reduction of approximately 10× compared with controls (41.1% ± 15.2%); (iii) finally, after demonstrating that mollusk HS is capable of preventing parasite binding under static and flow conditions, the effects of the compound on rosette disruption was investigated. We found that HS dose dependently disrupted rosettes, and at two high doses (100 and 1000 µg/mL), rosette formation was inhibited by approximately 100% relative to control. As shown in Fig. 3, HS disrupted rosettes at a rate similar to heparin. This rosette-disrupting activity was comparable with that reported for other sulfated polysaccharides, such as fucosylated chondroitin sulfate and curdlan sulfate.^9^ ^,^ ^10^ Indeed, it is well known that many glycosaminoglycans, including heparin and heparan sulfate, bind to the DBL1α domain of PfEMP-1, the major ligand involved in endothelial cytoadhesion, which can contribute to rosette formation.^19^

*In conclusion* - Here, we showed that HS from the bivalve mollusk *N. nodosus* effectively inhibited the development of the *P. falciparum* asexual blood stages in vitro. In addition, the mollusk HS disrupted rosetting and cytoadherence of Pf-iEs to CSA and ICAM-1 receptors expressed on endothelial cells, similar to heparin. The reversion of cytoadhesive phenotypes to ICAM-1, CSA, and rosetting is a desirable feature because these phenotypes are related to the severity of the malaria. Recently, it was shown that mollusk HS inhibited P-selectin activity,^12^ which can function as a receptor for PfEMP-^1(^ ^20^ and is known to contribute to malaria pathogenesis.^3^ Despite displaying comparable activity to heparin, HS does not cause relevant bleeding. Previously, we showed that mollusk HS has reduced anticoagulant activity compared with porcine heparin. Doses up to 10 mg/kg did not induce bleeding in rats or changes in plasma-activated partial thromboplastin times.^11^ The unique structural feature of mollusk HS relies on the presence of significant amounts of O-sulfate groups in glucuronic acid and glucosamine residues, not frequently found in their mammalian counterparts. No 3-O-sulfated glucosamine residues were detected, backing the observation that mollusk HS shows low antithrombin. This peculiar structure is unique in animal phyla and suggests the occurrence of sulfotransferases in the bivalve, with different substrate specificities of those observed in mammalians.^11^ ^,^ ^12^

However, there is no information about the effects of mollusk HS in humans. In our cytotoxicity assay, mollusk HS demonstrated no effect against non-infected erythrocytes at any concentration tested [Supplementary data (Fig. 2)], but a previous study showed reduced viability (by 35%) of BHK-21 (baby hamster kidney) cells in vitro at 100 µg/mL.^11^ Overall, the present study contributes to identification of a new heparin analog potentially useful as a safe adjunctive therapy for severe malaria.
