# Supplementary material for: A new heparan sulfate from the mollusk Nodipecten nodosus inhibits merozoite invasion and disrupts rosetting and cytoadherence of Plasmodium falciparum
Source: Mem Inst Oswaldo Cruz. 2019 Jun 6;114:e190088. doi: 10.1590/0074-02760190088 (PMC6555591; doi:10.1590/0074-02760190088)
Supplement: Supplementary file 1 [file 1678-8060-mioc-114-e190088-s.pdf]

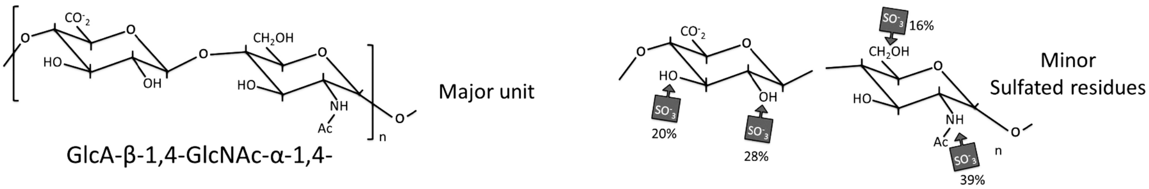

Fig. 1: major and minor disaccharide units of the mollusk heparan sulfate (HS). The major component is the non-sulfated disaccharide formed by ß-glucuronic acid 1,4 α-N-acetyl glucosamine. Variation in the degree of sulfation on the glucuronic acid and the N-acetyl glucosamine are present in the percentage indicated in the figure.

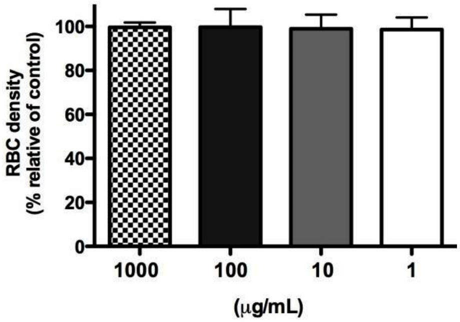

Fig. 2: mollusk heparan sulfate (HS) cytotoxic effect. Non-infected erythrocytes (niEs) were incubated during 48 h at 37ºC with increased concentrations of mollusk HS. As control, cells were incubated in RPMI medium only. Red blood cell density (RBCD) was determined as a percentage relative to control. Results are expressed as the mean of triplicates ± standard deviation (SD).

CC BY
